# Exploring a novel environment improves motivation and promotes recall of words

**DOI:** 10.3389/fpsyg.2014.00918

**Published:** 2014-08-20

**Authors:** Judith Schomaker, Marthe L. V. van Bronkhorst, Martijn Meeter

**Affiliations:** Department of Cognitive Psychology, VU University AmsterdamAmsterdam, Noord-Holland, Netherlands

**Keywords:** novelty, exploration, memory enhancement, episodic memory, dopaminergic mesolimbic system

## Abstract

Active exploration of novel environments is known to increase plasticity in animals, promoting long-term potentiation in the hippocampus and enhancing memory formation. These effects can occur during as well as after exploration. In humans novelty’s effects on memory have been investigated with other methods, but never in an active exploration paradigm. We therefore investigated whether active spatial exploration of a novel compared to a previously familiarized virtual environment promotes performance on an unrelated word learning task. Exploration of the novel environment enhanced recall, generally thought to be hippocampus-dependent, but not recognition, believed to rely less on the hippocampus. Recall was better for participants that gave higher presence ratings for their experience in the virtual environment. These ratings were higher for the novel compared to the familiar virtual environment, suggesting that novelty increased attention for the virtual rather than real environment; however, this did not explain the effect of novelty on recall.

## INTRODUCTION

Animal studies have consistently shown that exploration of a novel environment can promote long-term potentiation (LTP) in the hippocampus and specifically in the dentate gyrus, thereby improving memory encoding (Li et al., 2003; Straube et al., 2003a; Davis et al., 2004; Sajikumar and Frey, 2004; Sierra-Mercado et al., 2008). The modulatory effect of novelty on learning has been suggested to rely on the functional connections between the hippocampus and the substantia nigra/ventral tegmental area (SN/VTA), components of the mesolimbic dopaminergic system, by regulating the entry of new information into long-term memory (Lisman and Grace, 2005). When novelty is detected, a novelty signal from the hippocampus activates dopaminergic neurons in the SN/VTA. This results in dopamine being released in the hippocampus, where dopamine increases plasticity by enhancing LTP. Interestingly, increased plasticity not only occurs during exploration of the novel environment, but lasts up to 10 min afterwards (Li et al., 2003; Straube et al., 2003a; Kentros et al., 2004).

Direct evidence for a link between exploration of novel environments and increased plasticity comes from animal studies, but also in humans evidence for such a link has been found. Active as compared to passive exploration of a virtual environment (VE) has been shown to improve recall of allocentric spatial information (Voss et al., 2011; Plancher et al., 2012, 2013). Memory improvements have been observed for familiar items when these were presented in the context of novel scene stimuli (Bunzeck and Düzel, 2006). Similarly, passive exposure to novel scenes has been shown to improve recall of words presented in a task after exposure (Fenker et al., 2008). In this latter study either novel or previously familiarized scenes were presented for which indoor/outdoor judgments had to be made. After exposure to novel compared to familiar scenes recall and recollection on an unrelated word learning task were superior, whereas no such improvements were observed for familiarity-based recognition (Fenker et al., 2008). Also memory improvements have been found for items that stand out in the to-be-learned list, a benefit that may be novelty-related. For example, in the Von Restorff or distinctiveness effect memory formation is better for words presented in distinctive novel fonts than for words presented in a standard font (Von Restorff, 1933; Bruce and Gaines, 1976; Schmidt, 1985; Hunt, 1995; Dunlosky et al., 2000; Geraci and Rajaram, 2004; Geraci and Manzano, 2010; Rangel-Gomez and Meeter, 2013). Furthermore, the anticipation of a novel stimulus has been shown to enhance memory encoding for unexpected novel events (Wittmann et al., 2007). However, no studies so far have looked at whether active exploration of a novel environment promotes memory relative to familiar environments, or whether the effects of exploration extend to a subsequent learning task in humans.

**FIGURE 1:**
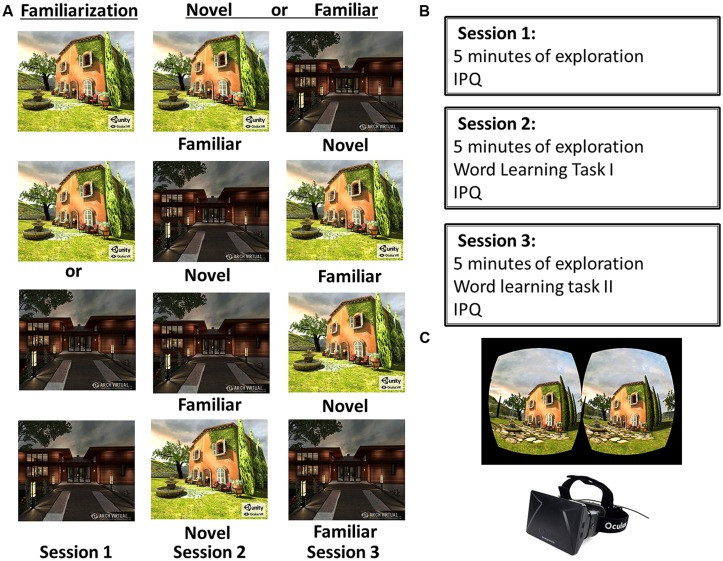
**Experimental design and apparatus. (A)** In session 1 participants were familiarized either with Tuscany or the Residential virtual environment (VE). In session 2 they either explored the familiarized VE (Familiar First) or the novel VE (Novel First). In the final session 3, participants in the Novel First condition explored the familiar VE, and participants in the Familiar First condition explored the novel VE. **(B)** Participants explored a VE and filled in the Igroup Presence Questionnaire (IPQ) in all sessions. In sessions 2 and 3 they also performed the word learning task. **(C)** Shows an example binocular display of the Tuscany VE, and the Oculus Rift head-mounted display.

In the present study, participants performed a word learning task either after exploring a previously familiarized or a novel VE from a first-person perspective in a within-subjects design (see **Figure 1**). In line with the findings above, we expected learning to be improved after exploring a novel VE, relative to a familiarized one. As was found by Fenker et al. (2008) and given that exploration of spatial novelty has been strongly associated with the hippocampus (Lisman and Grace, 2005; Jeewajee et al., 2008; Bast et al., 2009; Kaplan et al., 2012), we expected a learning benefit for free recall, which is thought to depend on the hippocampus (Yonelinas et al., 2002), and not for recognition, believed to rely mainly on the perirhinal cortex (Aggleton and Brown, 1999; Wan et al., 1999; Duzel et al., 2001; Fernandez et al., 2002; Yonelinas et al., 2002).

## MATERIALS AND METHODS

### PARTICIPANTS

Thirty-two participants naïve to the aims of the study participated in return for 15 or course credits. Two quit the experiment after the first session due to nausea/headaches, probably induced by exploring the VE, and two were excluded because they performed over two standard deviations below average on the word learning task. The remaining 28 participants (6 male; 25 right-handed; age 17–25 years; normal or corrected-to-normal vision) were included in the analyses. The study was performed in accordance with the ethical standards laid down in the 1964 Declaration of Helsinki and in accordance with the ethical committee of the faculty of Psychology and Education at the VU University Amsterdam, the Netherlands. Participants all signed written informed consent.

### STIMULI AND APPARATUS

**Figure 1** depicts the experimental design, procedures, and apparatus. Two existing VEs were used in this study: a Unity tech demo named Oculus Tuscany (we will refer to this VE as Tuscany^1^), and Architectural Visualization (Residential^2^). In both an indoor and outdoor scene could be explored. The VEs were presented using the Oculus Rift stereoscopic head-mounted display (Oculus VR), from a first-person perspective. The 7 inch LCD display has a field of view of 110° diagonal with a resolution of 640 × 800 per eye. Environmental sounds in the VEs were presented through sound-attenuating headphones.

The experimental task was presented on a LCD monitor (1680 × 1050 pixels; 120 Hz refresh rate). Participants were seated at a viewing distance of about 75 cm. During a study phase words were presented one by one in the center of the screen in a black 25 point font on a silver background [luminance CIE (0.34, 0.39), 61.60 cd/m^2^].

Two lists were constructed out of a longer list of 80 Dutch concrete nouns of 4–13 characters long, graciously provided by R. Zeelenberg, such that the average word-length was the same for the two lists. In addition the lists contained the same number of words from different semantic categories (e.g., animals, food, locations, body parts). 40 words were presented during a study phase, and 40 served as lures during a recognition phase. Per phase the same words were used for all participants.

### PROCEDURE

On three separate days within a 5-day span participants actively explored one of two VEs (Tuscany; Residential) for 5 min. Participants either explored Tuscany or Residential on the first day (session 1), counterbalanced between subjects. In session 2 participants either explored the same familiar or the other novel VE (Familiar first; Novel first), and in session 3 the other VE (either Novel second; Familiar second) – order again counterbalanced between subjects. Note, in this within-subjects design all participants thus explored a familiar and a novel VE.

Before exploration participants were told how to navigate through the VE, using the mouse to indicate direction and using the keyboard arrows to move forward. Head movements were tracked and displayed accordingly, to create a sense of realism and to increase immersion. Participants were instructed to “keep moving” and to explore the entire VE. After 5 min exploration was terminated. In a pilot it was found that 5 min was sufficient to exhaustively explore the VEs.

After exploration in session 1 participants filled in the Igroup Presence Questionnaire (IPQ; Schubert et al., 2001) to measure the subjective experience of spatial presence, involvement in and realism of the VE. In sessions 2 and 3 participants performed the word learning task directly after exploration of the VE. Participants were instructed to pay attention to the words that were presented and to remember them. During the study phase in total 40 words were presented for a duration of 2000 ms each. The words were separated by a central fixation cross that was presented for 1000 ms. After the study phase, participants could type in the words that they remembered during a free recall phase. After the recall phase participants performed a recognition test. All 40 words from the study phase were presented intermixed with 40 lure words in a randomized sequence. Participants indicated for every word whether it was presented during the study phase (old: press “m”) or not (new: press “x”). In both sessions 2 and 3, after completing the word learning task, participants again filled in the IPQ with respect to their experience in the VE that day. The learning task was performed in about 15 min.

Recall performance was calculated as the proportion of correctly remembered items. Words containing errors (<2 typos) were coded as correct, unless the answer resulted in another meaningful word (for example “bow” instead of “boy” would be coded as incorrect, but “boyyy” would be coded as correct). For recognition memory performance hits were defined as the correctly recognized words, and false alarms as the new words that were falsely identified as words presented in the study phase. Recognition memory performance was defined by the sensitivity measure *d^′^*. In addition, the response bias measure β was calculated.

The effects of exploring a familiar or novel environment on memory performance were investigated with separate 2 × 2 × 2 repeated measures ANOVAs with the within-subjects factor Novelty (Novel; Familiar) and which type of VE was explored in session 2, VE First (Novel; Familiar), and which VE was familiarized, VE Familiar (Tuscany; Residential) as between-subjects factors.

The IPQ scores after exploration of a novel and a familiar VE were compared using a paired-samples *t*-test. To further investigate the effects of presence on memory the group was divided into low- and high-scorers using a median split on basis of the mean IPQ scores over the three sessions. Additional 2 × 2 repeated-measures ANOVAs were performed with Novelty (Novel; Familiar) as a within-subjects factor and Presence (Low; High) as a between-subjects factor, investigating the effects on hits and false alarms during recognition, and on recall. As a follow-up, linear regression analysis was performed with recall after exploring a novel minus a familiar VE (novelty recall benefit) as the critical variable and IPQ ratings after exploring a novel minus a familiar VE (presence difference score) as the predictor variable.

## RESULTS

**FIGURE 2:**
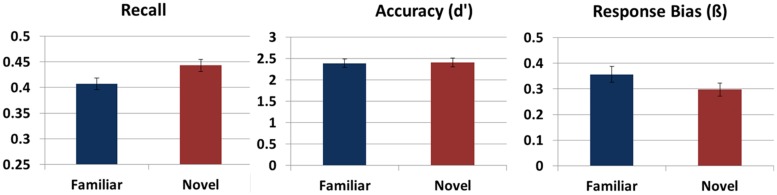
**Memory performance on the word learning task.** Recall memory, accuracy as defined by *d’* on the recognition test, and response bias on the recognition test after exploring a novel and familiar environment. Error bars reflect standard errors.

**Figure 2** shows average performance on the word learning task after exploring both a novel and familiar environment. Recall was better after participants explored the novel versus the familiar VE, *F*(1,24) = 4.01, *p* = 0.046, 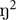 = 0.16. Whether participants explored the novel or familiar VE in the first session did not affect recall (*F* < 1), nor did it matter which VE was familiarized, *F*(1,24) = 1.45, *p* = 0.240, 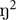 = 0.06. There was a trend for an interaction between Novelty and which VE was familiarized, *F*(1,24) = 3.19, *p* = 0.087, 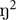 = 0.12, with effects of novelty being stronger when Tuscany than when Residential was familiarized. Other factors did not interact with Novelty (*p* > 0.554).

Whether the novel or familiar VE was explored or which type of VE was explored first did not affect sensitivity during recognition (*F* < 1). There was a trend for higher performance for the participants that were familiarized with Tuscany rather than Residential, *F*(1,24) = 4.10, *p* = 0.054, 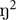 = 0.15. No interactions with Novelty were found (*F* < 1). In addition, no differences in response bias were found after exploring a novel or familiar VE (*F* < 1). Novelty did not interact with which VE was familiarized (*F* < 1), nor with whether participants first explored the novel or familiar VE, *F*(1,24) = 1.47, *p* = 0.237, 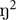 = 0.06.

**FIGURE 3:**
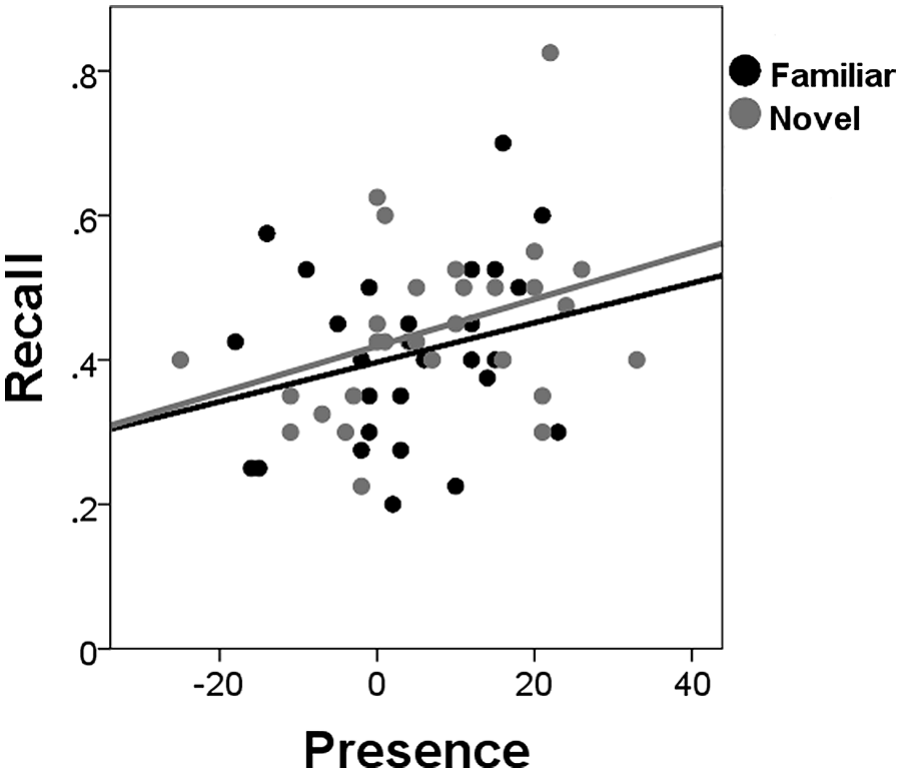
**Recall for low and high presence.** The proportion of correctly remembered words after exploration of the novel and familiar environments relative to the respective IPQ scores (Presence).

Presence ratings (IPQ) were higher after exploration of the novel (mean = 7.32; SD = 13.41) than of the familiar VE (mean = 3.79; SD = 11.44), *t*(27) = 2.11, *p* < 0.045. In addition, recall memory was better for participants who gave high compared to low presence ratings, *F*(1,26) = 4.96, *p* = 0.035, 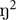 = 0.16. Presence did not interact with Novelty, *F*(1,20) = 2.29, *p* = 0.146, 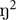 = 0.10. No effects of presence were found for recognition (neither hits nor false alarms; *F* < 1). To investigate whether novelty’s effects on recall were the result of higher presence ratings in the novel VE, linear regression analysis was performed for the novelty recall benefit with the presence difference score as the predictor. The presence difference score was not related to the novelty recall benefit, β = -0.002, *t*(27) = -0.865, *p* = 0.395, and only marginally lowered the effect of novelty on recall, suggesting that presence and novelty independently affected recall. **Figure 3** shows the recall scores after exploration of a novel or familiar environment and the corresponding IPQ presence scores.

## DISCUSSION

The effect of exploring of a novel versus a familiar environment was investigated in humans using virtual reality environments and performance on a subsequent word learning task. Recall was enhanced for words learned after exploration of a novel compared to a familiar environment. No such effect was found for recognition. Recall is believed to rely strongly on the hippocampus, while recognition depends more on the perirhinal cortex and thalamus (Aggleton and Brown, 1999; Wan et al., 1999; Fernandez et al., 2002). Therefore, these findings are in line with the Lisman and Grace’s (2005) model that proposes that a novelty signal from the hippocampus may promote subsequent memory encoding by dopamine release from the SN/VTA projecting back to the hippocampus. Familiarity-based recognition does not strongly rely on the hippocampus, which means that the Lisman and Grace (2005) model does not predict an effect of novelty on recognition. Indeed no effects were found on recognition. In contrast, Bunzeck and Düzel (2006) reported enhanced recognition memory for familiar items when these were presented in a block in which also novel stimuli were presented, relative to when they were presented among even more familiar items. Their finding is difficult to compare to the present study’s results, since their measure of recognition was corrected hit rate, subtracting false alarms from hits. This procedure does not fully disentangle changes in accuracy from criterion shifts, which leaves open the possibility that their results reflect a shift in the response criterion rather than improvements in memory.

Exposure to novelty has been reliably shown to improve memory in non-human animals (Li et al., 2003; Straube et al., 2003a; Davis et al., 2004; Sajikumar and Frey, 2004; Sierra-Mercado et al., 2008), as has exploration (Li et al., 2003; Straube et al., 2003a). In humans, memory is better for distinctive items in a list than for non-distinctive items. This so-called Von Restorff effect (Von Restorff, 1933) has sometimes been taken to reflect an effect of novelty on learning (Kishiyama et al., 2004, 2009), though some authors have argued that other mechanisms are responsible for this effect (Bruce and Gaines, 1976; Schmidt, 1985; Hunt, 1995; Dunlosky et al., 2000; Geraci and Rajaram, 2004; Rangel-Gomez et al., 2012; Rangel-Gomez and Meeter, 2013). Our study, and that of Fenker et al. (2008) are the only ones that report an effect in humans similar to those reported in non-human animals: temporally extended memory enhancements after exposure to novelty. In our study, exploration of novel environments improved learning in an unrelated task that was on average 10 min after the exploration phase. Temporally extended novelty-induced memory enhancements may be related to the mesolimbic dopamine system. Increased dopaminergic drive in the hippocampus may promote encoding of new information especially after novelty detection and has been associated with a oscillatory theta state in the hippocampus (Lisman and Otmakhova, 2001). Indeed, activation of dopaminergic D1/D5 receptors during exploration of a novel environment has been found to lower the threshold for LTP and learning in the hippocampus in rats (Li et al., 2003). Also in humans, novelty triggers dopamine release in the SN/VTA, which results in better memory performance (Schott et al., 2004; Wittmann et al., 2007; Apitz and Bunzeck, 2013; Eckart and Bunzeck, 2013). Dopamine is known to result in increased plasticity in the hippocampus (Chen et al., 1999; Lisman et al., 2011), and the effects of novelty on dopamine release are known to affect plasticity 10 min later (Li et al., 2003; Straube et al., 2003a; Kentros et al., 2004).

Long-lasting effects may also partly be mediated by motivation. Dopamine release may result in improved motivation by eliciting an “exploration bonus” (Kakade and Dayan, 2002; Lisman and Grace, 2005; Bunzeck and Düzel, 2006; Knutson and Cooper, 2006; Bunzeck et al., 2007, 2012; Krebs et al., 2009, 2011; Duzel et al., 2010; Guitart-Masip et al., 2010), which may also have contributed to enhanced recall memory. This is supported by the fact that recall was higher for participants who reported a stronger sense of presence, and that participants gave higher presence ratings for the novel than for the familiar environment. Presence has been linked to attention allocated to the virtual rather than the real world (Darken et al., 1999), and may thus correlate with how motivated participants were during the exploration. This could possibly have contributed to the novelty-induced memory enhancements. However, since differences in presence in the novel and familiar VE were not related to the novelty recall benefit it cannot explain the novelty effect. Presence thus promotes memory via a relatively independent mechanism.

Alternatively, the observed learning benefits may be caused by increased arousal as mediated by noradrenergic activity. Exploration of novel environments results in arousal by increasing noradrenergic activity (Moser et al., 1994; Sara et al., 1994; Vankov et al., 1995; Kitchigina et al., 1997; Straube et al., 2003b), enhancing LTP and memory formation (Klukowski and Harley, 1994; Cahill and McGaugh, 1998). Also the cholinergic system has been linked to novelty’s effect on learning (Hasselmo, 1999; Ranganath and Rainer, 2003; Meeter et al., 2004; Barry et al., 2012); acetylcholine has been shown to be present in cortex in higher levels during exploration of a novel rather than a familiar environment (Giovannini et al., 2001). Noradrenergic, cholinergic and dopaminergic neuromodulatory systems could each underlie the novelty-induced memory enhancements, alone or in combination; direct physiological evidence linking these systems to memory performance is still lacking.

In conclusion, our findings suggest that active exploration of a novel environment can promote memory encoding also in humans, and that these effects linger for some time after exploration. In addition, individual differences and fluctuations in the amount of attention allocated to the environment during exploration also affect learning in the later memory task.

## Conflict of Interest Statement

The authors declare that the research was conducted in the absence of any commercial or financial relationships that could be construed as a potential conflict of interest.

## Footnotes

1 http://www.riftenabled.com/admin/app/17

2 http://www.riftenabled.com/admin/app/268
